# Sentiment Analysis of Insomnia-Related Tweets via a Combination of Transformers Using Dempster-Shafer Theory: Pre– and Peri–COVID-19 Pandemic Retrospective Study

**DOI:** 10.2196/41517

**Published:** 2022-12-27

**Authors:** Arash Maghsoudi, Sara Nowakowski, Ritwick Agrawal, Amir Sharafkhaneh, Mark E Kunik, Aanand D Naik, Hua Xu, Javad Razjouyan

**Affiliations:** 1 Department of Medicine Baylor College of Medicine Houston, TX United States; 2 Department of Management, Policy, and Community Health University of Texas School of Public Health The University of Texas Health Science Center at Houston Houston, TX United States; 3 School of Biomedical Informatics The University of Texas Health Science Center at Houston Houston, TX United States

**Keywords:** COVID-19, coronavirus, sleep, Twitter, natural language processing, sentiment analysis, transformers, Dempster-Shafer theory, sleeping, social media, pandemic, effect, viral infection

## Abstract

**Background:**

The COVID-19 pandemic has imposed additional stress on population health that may result in a change of sleeping behavior.

**Objective:**

In this study, we hypothesized that using natural language processing to explore social media would help with assessing the mental health conditions of people experiencing insomnia after the outbreak of COVID-19.

**Methods:**

We designed a retrospective study that used public social media content from Twitter. We categorized insomnia-related tweets based on time, using the following two intervals: the prepandemic (January 1, 2019, to January 1, 2020) and peripandemic (January 1, 2020, to January 1, 2021) intervals. We performed a sentiment analysis by using pretrained transformers in conjunction with Dempster-Shafer theory (DST) to classify the polarity of emotions as *positive*, *negative*, and *neutral*. We validated the proposed pipeline on 300 annotated tweets. Additionally, we performed a temporal analysis to examine the effect of time on Twitter users’ insomnia experiences, using logistic regression.

**Results:**

We extracted 305,321 tweets containing the word *insomnia* (prepandemic tweets: n=139,561; peripandemic tweets: n=165,760). The best combination of pretrained transformers (combined via DST) yielded 84% accuracy. By using this pipeline, we found that the odds of posting negative tweets (odds ratio [OR] 1.39, 95% CI 1.37-1.41; *P*<.001) were higher in the peripandemic interval compared to those in the prepandemic interval. The likelihood of posting negative tweets after midnight was 21% higher than that before midnight (OR 1.21, 95% CI 1.19-1.23; *P*<.001). In the prepandemic interval, while the odds of posting negative tweets were 2% higher after midnight compared to those before midnight (OR 1.02, 95% CI 1.00-1.07; *P*=.008), they were 43% higher (OR 1.43, 95% CI 1.40-1.46; *P*<.001) in the peripandemic interval.

**Conclusions:**

The proposed novel sentiment analysis pipeline, which combines pretrained transformers via DST, is capable of classifying the emotions and sentiments of insomnia-related tweets. Twitter users shared more negative tweets about insomnia in the peripandemic interval than in the prepandemic interval. Future studies using a natural language processing framework could assess tweets about other types of psychological distress, habit changes, weight gain resulting from inactivity, and the effect of viral infection on sleep.

## Introduction

The COVID-19 pandemic has imposed excessive stress on the world population [1,2] through financial instability, unemployment, social isolation, and a lack of social activities [3]. Prior studies established the association between this stress and sleep disturbances [4-6]. Additionally, due to the pandemic, restrictions such as social distancing have resulted in the increase of certain digital behaviors, including distance learning, web-based meetings, web-based shopping, and social media usage [7-9]. The rise in the usage of social media platforms, like Twitter, provides researchers with a new source of data for screening public behavior.

Several studies have reported the impact of the COVID-19 pandemic on sleep quality and mental health [10-17]. However, these studies were limited to small databases, data gathered through questionaries, or both, and they lacked a comparison group. For instance, one study used Twitter to report the effect of the COVID-19 pandemic on the sleep quality of pregnant women based on 192 tweets [18]. The sentiment analysis of social media content is a challenging task, since such texts are unstructured, brief, informal, and casual; are prone to mistakes in dictation and grammar; and are noisy (emojis, hashtags, URLs, etc); and they entail ambiguities, such as polysemy [19]. Therefore, using artificial intelligence and machine learning tools and techniques may prove to be beneficial for tackling these challenges. Among these tools are advanced, analytical natural language processing (NLP) algorithms called *transformers* [19-26]. They are newly proposed tools and extensions to previous versions of a deep artificial neural network—recurrent neural networks—for language modeling and language encoding.

We hypothesized that using NLP to explore social media could help with assessing the mental health conditions of people experiencing insomnia after the outbreak of the COVID-19 pandemic. Mental health was defined by measuring negative sentiment, using NLP algorithms on publicly available data from Twitter. We designed a sentiment analysis pipeline based on pretrained transformers’ architectures. The output of transformers was combined via Dempster-Shafer theory (DST; theory of belief) to achieve higher accuracy in the recognition of sentiments. The performance of this model was verified for accuracy by using a manually annotated data set. Subsequently, using this pipeline, we analyzed and compared the sentiments inherent in insomnia-related tweets that were posted within 1 year before the COVID-19 pandemic outbreak (prepandemic) and within 1 year during the pandemic (peripandemic). We also compared the results of the sentiment analysis of the tweets in terms of tweets’ posting times (ie, temporal analysis; before midnight vs after midnight).

## Methods

### Study Design and Data Collection

**Figure 1:**
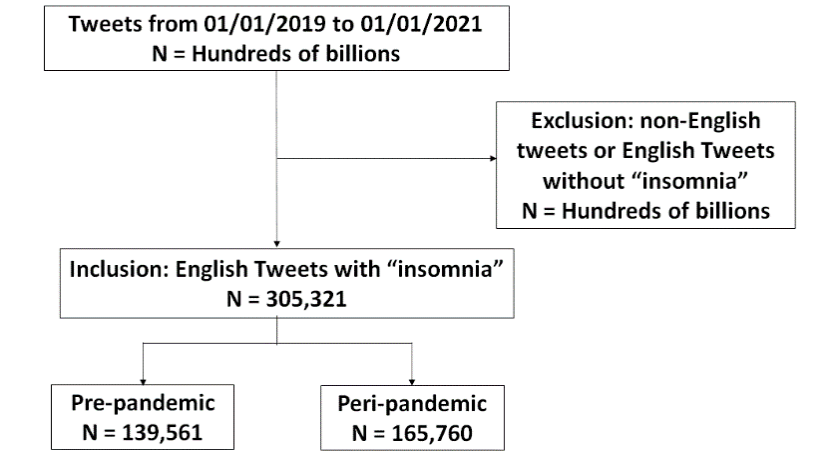
STROBE (Strengthening the Reporting of Observational Studies in Epidemiology) diagram.

This retrospective pilot study examined tweets that were posted in the 2019 calendar year (prepandemic interval) and the 2020 calendar year (peripandemic interval). We collected publicly available English tweets by using the Twitter application programming interface, which allowed us to collect tweets by matching keywords (ie, *insomnia*). The tweets were classified into two groups—prepandemic (January 1, 2019, to January 1, 2020) and peripandemic (January 1, 2020, to January 1, 2021) tweets—based on the posting dates and times. The inclusion criteria for tweets were that they must contain the word *insomnia* and be in English. Therefore, all non-English tweets and English tweets without the keyword *insomnia* were excluded (Figure 1). The data extracted from included tweets were used for sentiment analysis and for sentiment annotation.

### Sampling Strategy and Annotation

To determine the minimum required sample size for the NLP algorithm performance measurement, we used the exact power calculation method [27]. We assumed that for an effect size of 0.3, an α of .05, a power of 80, and 5 *df*, 143 notes would be required. However, our team of annotators reviewed 300 randomly selected notes.

To verify the performance of the models in predicting the tweets’ sentiments, we randomly chose 300 tweets from the data extracted (according to the *Study Design and Data Collection* section) and manually annotated them into the positive, negative, and neutral categories. Two nonnative English speakers with International English Language Testing System scores of ≥7 annotated the tweets. A third senior nonnative English speaker served as a final judge to adjudicate disagreements. We used the Cohen κ [28] parameter to measure the interrater reliability between annotators.

### Developing a Sentiment Analysis Pipeline for Tweets

#### Sentiment Analysis Pipeline Overview

**Figure 2:**
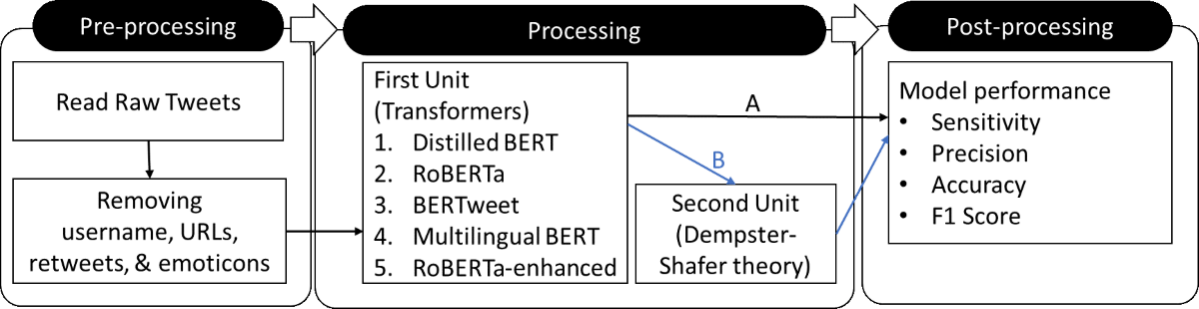
The machine learning natural language processing algorithm pipeline. (A) We calculated the performance of each transformer separately. (B) The output of transformers was combined, using the Dempster-Shafer theory to make the final decision. BERT: Bidirectional Encoder Representations From Transformers; RoBERTa: Robustly Optimized Bidirectional Encoder Representations From Transformers Pretraining Approach.

We devised an algorithm that had the following three steps: preprocess, process, and postprocess. In the preprocess step, we prepared the tweets for the process step by removing special characters, URLs, and hashtags. The process step consisted of 2 units. The first unit performed sentiment classification (ie, positive, negative, and neutral), using multiple models. The second unit used DST to combine the output from several models (ie, those from the previous step) to provide a more accurate prediction. Finally, in the postprocess step, we quantified the sentiment analysis performance of different models. These steps are discussed in more detail in the following sections and in Figure 2.

#### Preprocessing

Raw data scraped from Twitter contain irrelevant attributes (eg, usernames, URLs, retweets, emoticons, etc). The purpose of preprocessing was to filter undesired text content and obtain relevant parts of the tweets.

#### Process

The process step consisted of the following two units: NLP-based sentiment analysis classifiers and DST, which was used to combine the classifiers’ outputs.

##### First Unit: Transformers

**Figure 3:**
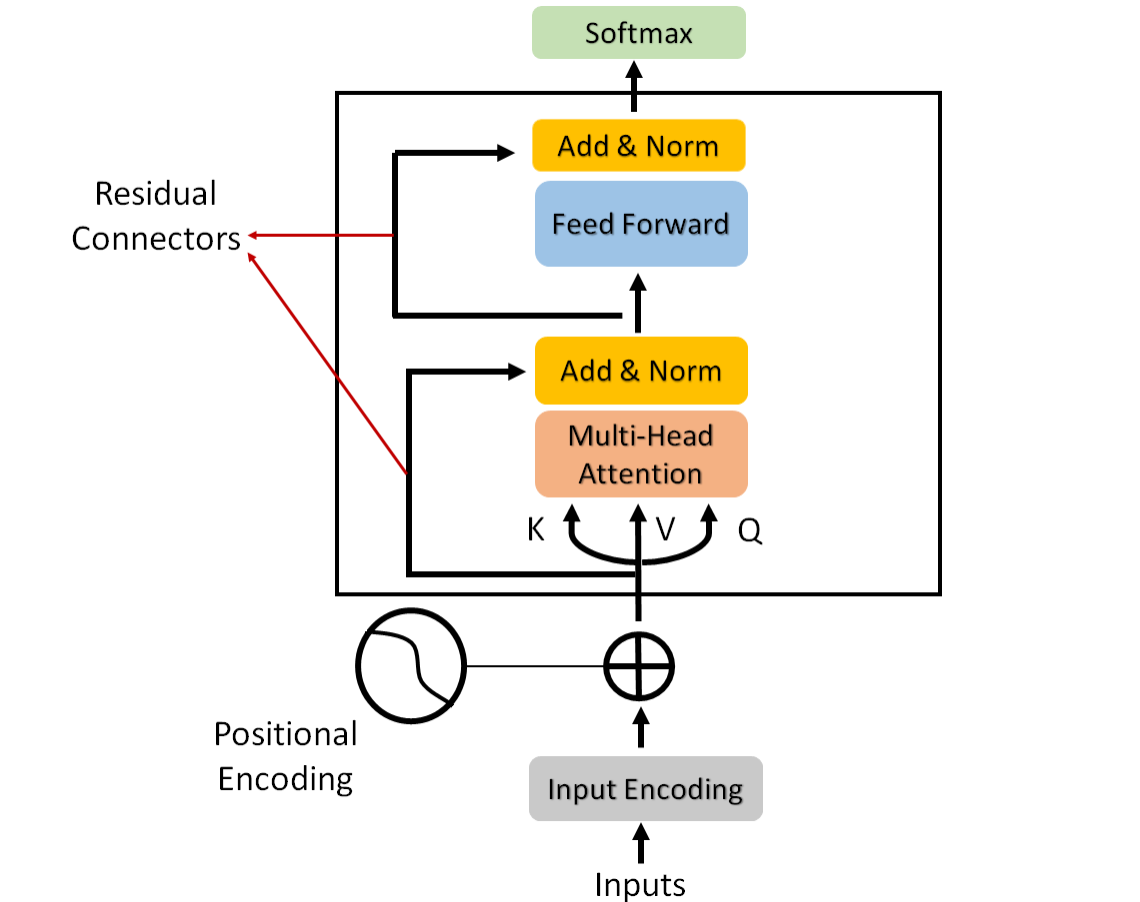
Classification procedure with a transformer.

To perform the sentiment analysis on tweets, we took advantage of transformers, which are the new generation of deep artificial neural networks (also known as *recurrent neural networks*) that were introduced for machine translation [29] and were constructed by stacking transformer units on top of each other. They comprise two main blocks—an encoder and a decoder. The encoder is used for classification and inference, and the decoder is mainly used for language modeling; the complete architecture is used for machine translation [30]. A typical encoder of a transformer is shown in Figure 3 (Multimedia Appendix 1 provides a brief theory of transformers).

A total of 5 different pretrained transformer-based models for the sentiment analysis of tweets were used. The five pretrained models provided by the Hugging Face AI community are as follows:

1. Distilled Bidirectional Encoder Representations From Transformers (BERT) [31], which was fine-tuned on the Stanford Sentiment Treebank v2 database [32]. Knowledge distillation [33,34] was used to reduce the size of a BERT model by 40% while preserving 97% of its language understanding capabilities and making it 60% faster.
2. Robustly Optimized BERT Pretraining Approach (RoBERTa) [35] for sentiment analysis, which was trained on around 58 million tweets. The RoBERTa model was based on the BERT structure; however, it was pretrained on not only the data that BERT was trained on (BookCorpus [34,36] and English Wikipedia; around 3.3 billion words) but also a news data and stories database [37]. RoBERTa was fine-tuned on 58 million tweets for sentiment analysis.
3. BERTweet [38], which was trained based on the RoBERTa pretraining procedure and pretrained on 850 million English tweets.
4. The multilingual BERT-based model, which was fine-tuned for sentiment analysis on product reviews in the following six languages: English, Dutch, German, French, Spanish, and Italian. It predicts the sentiment of a review by using stars (between 1 and 5 stars); 3 stars are considered neutral, <3 are considered negative, and ≥4 are considered positive.
5. The RoBERTa [35] model that was fine-tuned on 15 data sets from diverse text sources to enhance generalization across different types of texts (reviews, tweets, etc).

##### Second Unit: DST

To increase the performance of the transformer models discussed in the *First Unit: Transformers* section, we used DST [39,40], which has the ability to combine evidence from different experts. We let *Θ* = {*θ_1_*, *θ_2_*,…, *θ_l_*} be a finite set of possible hypotheses. This set is referred to as the *frame of discernment*, and its powerset is 2^Θ^. We defined a function, *m(.)*, called a *basic belief assignment*, which maps every subset *η* of Θ to a value ranging from 0 to 1 and satisfies the following conditions:

*m(ϕ)*=0 **(1)**

and



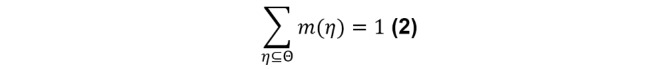



A subset ζ for which *m*(*η)* is >0 is called a *focal element*. We defined another function called *the belief function*, *bel(.)*, which assigns a value ranging from 0 to 1 to every nonempty subset ζ of Θ and is defined as follows:



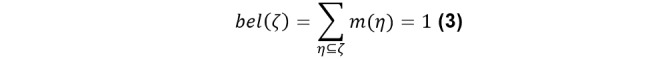



Given the above functions, we defined the combination rule. We assumed 2 basic belief assignments, *m_1_(.)* and *m_2_(.)*, for belief functions *bel_1_(.)* and *bel_2_(.)* and let *η*_j_ and ζ_k_ be focal elements of *bel_1_* and *bel_2_*, respectively. *m_1_(.)* and *m_2_(.)* were then combined to obtain the belief mass committed to *ϑ*⊆Θ, according to the following combination (ie, orthogonal sum formula):



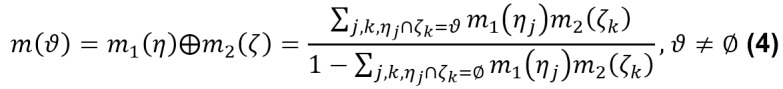



where the denominator is essential for normalization.

#### Postprocess: Model Evaluation

To evaluate the performance of the models discussed in the *First Unit: Transformers* section, evaluation metrics—sensitivity, precision, accuracy, and *F*_1_ score—extracted from the confusion matrix were used in this study and were calculated by using the following equations [41]:



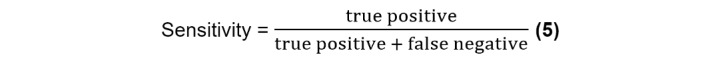





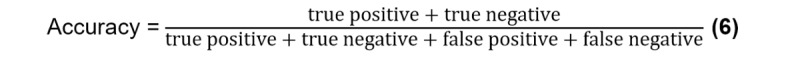





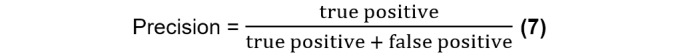





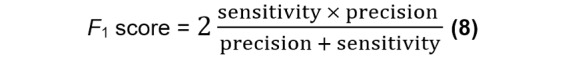



### Statistical Analysis

After performing the sentiment analysis and dividing the data into the negative, positive, and neutral categories, the categorical characteristics (number of negative, positive, and neutral tweets) of these tweets were analyzed by using the chi-square test and odds ratios (ORs). *P* values with a significance level of <.05, 95% CIs, and *z*-statistics were reported. Data management was performed with Python 3.8 [42], and the analysis was performed with SPSS version 27 (IBM Corporation).

### Temporal Analysis

We also investigated the chronology of insomnia-related tweets by examining the overall hourly number of tweets. We extracted the posting times of tweets with a negative sentiment. The daily hours were then categorized into the following two time spans: before midnight (1 PM to midnight) and after midnight (1 AM to noon). We calculated the percentage of negative tweets in each interval and used a logistic regression analysis to compare the odds of posting negative tweets before and after midnight.

## Results

### Characteristics of Tweets

We retrieved 305,321 tweets that contained the word *insomnia* and were posted in the prepandemic and peripandemic periods. Of these, 139,561 were posted in the prepandemic period, and 165,760 (an 18.7% increase) were posted in the peripandemic interval. The tweets’ length (number of words) was approximately the same between these two time periods (prepandemic: mean 26.3, SD 13.7 words; peripandemic: mean 29.3, SD 13.7 words). The number of tweet interactions, defined as the summation of the number of likes, retweets, and replies, did not differ significantly (*P*<001) (prepandemic: mean 6.2, SD 171.8 interactions; peripandemic: mean 5.4, SD 100.6 interactions).

### Annotation

Of the 300 tweets that were annotated by the two reviewers, 167 (55.7%) were classified as negative, 102 (34%) were classified as neutral, and 31 (10.3%) were classified as positive. The interrater reliability reached 0.55 (95% CI 0.44-0.69).

### Sentiment Analysis Pipeline Performance

In Table 1, we report the accuracy of the five models that were pretrained on 300 annotated tweets. Model 1—Distilled BERT—had the best performance (80.3%). After combining the models by using the DST approach, we observed that combining models 1, 2, 3, and 5 resulted in the highest performance (84%; Table 1).

Since Distilled BERT (model 1) showed the best performance for single-model classification, and to better understand how DST improves the performance of the pipeline, we analyzed the evaluation metrics of this model alongside those of the best combination of models (ie, the one reported in Table 1), which showed overall better performance for all 3 categories of sentiments (Table 2).

**Table 1:** Comparison of the performance of the models used to analyze the 300 annotated tweets.

Models			Accuracy (%)
**Individual models**			
	Model 1 (Distilled BERT^a^) [31]	80.3	
	Model 2 (RoBERTa^b^) [35]	52.7	
	Model 3 (BERTweet^c^) [38]	53	
	Model 4 (BERT-multilingual) [35]	49.3	
	Model 5 (fine-tuned RoBERTa) [35]	45.3	
**Combined models based on Dempster-Shafer theory [39,40]**			
	Model 1+model 2+model 3	81	
	Model 1+model 2+model 3+model 5	84	
	Model 1+model 5	77.2	
	Model 1+model 2+model 3+model 4+model 5	81.7	

^a^BERT: Bidirectional Encoder Representations From Transformers [31].

^b^RoBERTa: Robustly Optimized Bidirectional Encoder Representations From Transformers Pretraining Approach [35].

^c^BERTweet is a Robustly Optimized Bidirectional Encoder Representations From Transformers Pretraining Approach model that was trained on 850 million English tweets [38].

**Table 2:** Comparison of the performance of the individual model—Distilled Bidirectional Encoder Representations From Transformers—and the combined model based on Dempster-Shafer theory in identifying each sentiment class (positive, neutral, and negative).

Sentiment	Sensitivity (%)			Precision (%)			*F*_1_ score			Accuracy (%)	
	Individual model^a^	Combined model^b^	Individual model		Combined model	Individual model		Combined model	Individual model		Combined model
Negative	92.8	93.4	77.9		81.7	84.7		87.1	81.3		84.6
Neutral	72.5	77.5	98.7		98.8	83.6		86.8	90.3		91.3
Positive	38.7	54.8	46.2		58.6	42.1		56.6	89		92

^a^The individual model is Distilled Bidirectional Encoder Representations From Transformers [31].

^b^The combined model is the combination of Distilled Bidirectional Encoder Representations From Transformers (BERT) [31], Robustly Optimized BERT Pretraining Approach (RoBERTa) [35], BERTweet [38], and fine-tuned RoBERTa [35].

### Sentiment Analysis

**Figure 4:**
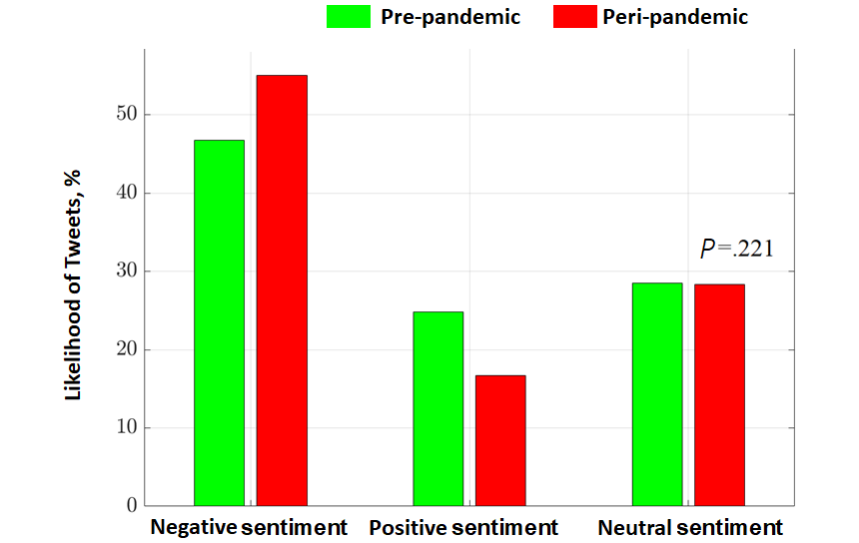
Likelihood of posting negative, positive, and neutral tweets in the prepandemic and peripandemic periods. *P<.001.

The results of the best combined model for sentiment analysis that was applied to all of the tweets are shown in Table 3. We observed a higher likelihood of posting negative tweets during the peripandemic period (91,242/165,760, 55%) compared to that during the prepandemic period (65,164/139,561, 46.7%). Accordingly, we observed a lower likelihood of posting positive tweets during the peripandemic period (27,621/165,760, 16.7%) compared to that during the prepandemic period (34,633/139,561, 24.8%). We also observed the same likelihood of posting neutral tweets during the peripandemic and postpandemic periods (Figure 4). We reported 39% higher odds of posting negative tweets during the peripandemic period compared to those during the prepandemic interval (OR, 1.39; 95% CI, 1.37-1.41, *P*<.001; Table 3).

**Table 3:** Characteristics of negative and positive prepandemic (calendar year 2019) tweets and peripandemic (calendar year 2020) tweets.

Tweet sentiment	Total tweets (N=305,321), n (%)	Prepandemic tweets (n=139,561), n (%)	Peripandemic tweets (n=165,760), n (%)	Prepandemic vs peripandemic		
				*P* value	*z*-statistic	Odds ratio (95% CI)
Negative tweets	156,406 (51.3)	65,164 (46.7)	91,242 (55)	<.001	45.94	1.39 (1.37-1.41)
Positive tweets	62,254 (20.4)	34,633 (24.8)	27,621 (16.7)	<.001	55.402	0.60 (0.59-0.61)
Neutral tweets	86,661 (28.3)	39,764 (28.5)	46,897 (28.3)	.22	1.22	0.99 (0.97-1.00)

### Temporal Analysis

**Figure 5:**
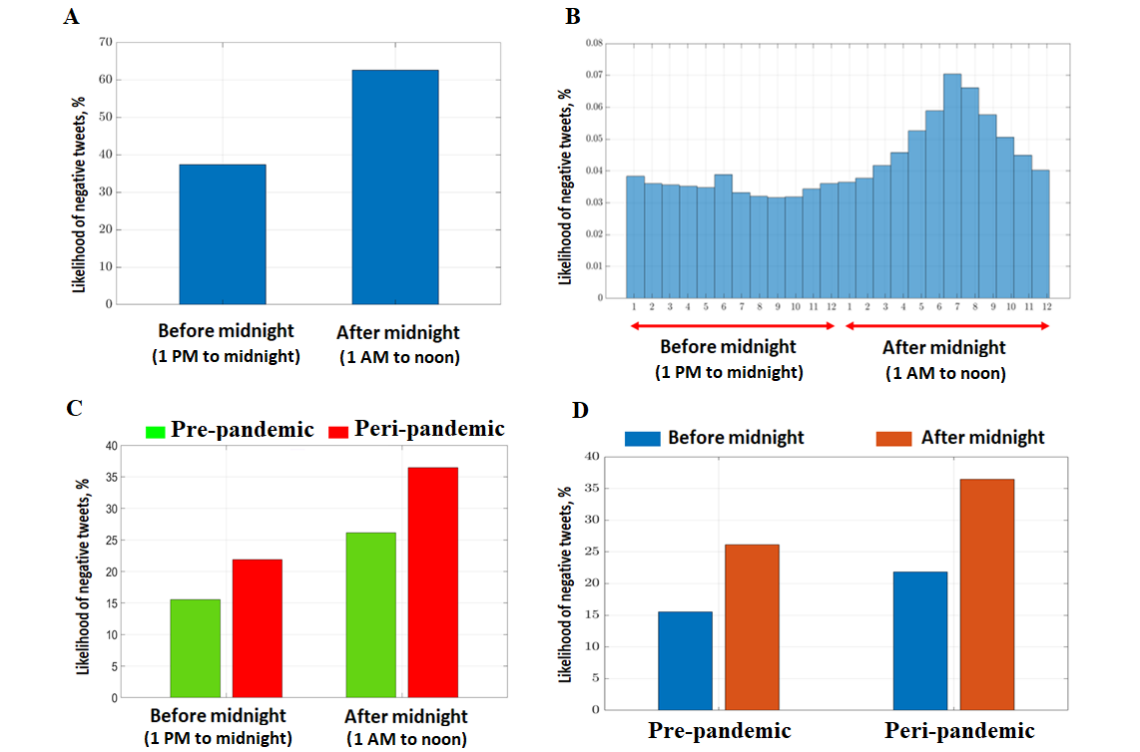
Temporal analysis of tweets. (A) Percentage of negative tweets posted before midnight (1 PM to midnight) and after midnight (1 AM to noon). (B) Hourly distribution of negative tweets. (C) Comparison of the likelihood of posting negative tweets before midnight (1 PM to midnight) and after midnight (1 AM to noon) for the prepandemic and peripandemic periods. (D) Comparison of the likelihood of posting negative tweets before midnight (1 PM to midnight) and after midnight (1 AM to noon) for the prepandemic and peripandemic periods.

The likelihood of posting negative tweets after midnight was higher than that before midnight (OR 1.21, 95% CI 1.19-1.23; *P*<.001; Figure 5A). An increasing trend was observed during after-midnight intervals when compared to before-midnight intervals, according to the hourly distribution of negative tweets (Figure 5B). The odds of posting negative tweets before midnight during the peripandemic period were 15% higher than those during the prepandemic period (OR 1.15, 95% CI 1.12-1.18; Figure 5C), while the odds posting negative tweets after midnight was 60% higher during the peripandemic period (OR 1.60; 95% CI 1.57-1.63; *P*<.001; Figure 5C). In the prepandemic period, the odds of posting negative tweets were 2% higher after midnight compared to those before midnight (OR 1.02, 95% CI 1.00-1.07; *P*=.008; Figure 5D); however, they were 43% higher in the peripandemic period (OR 1.43, 95% CI 1.40-1.46; *P*<.001; Figure 5D). The results of a quarterly (3-month) analysis of tweet sentiments for the prepandemic and peripandemic intervals are presented in Table S1 and Figure S2 in Multimedia Appendix 2.

## Discussion

### Principal Findings

In this retrospective cohort study, we showed that NLP tools can monitor population health by using the sentiments expressed on a publicly available platform, such as Twitter, as a surrogate measure of public awareness and perception. We observed that the COVID-19 pandemic was negatively associated with a change in insomnia-related self-report tweets. We designed a novel NLP pipeline for sentiment analysis that was based on a combination of pretrained transformers (combined via DST; ie, theory of belief). By using this basis, which was validated on manually annotated tweets, we detected more negative tweets during the peripandemic interval than those detected during the prepandemic interval among people reporting insomnia on Twitter.

First, we developed a novel machine learning–based pipeline to analyze emotions. To verify the performance of models, we manually annotated 300 tweets. The κ analysis showed an agreement of 55% among different raters. This is not a very strong agreement, and this could have resulted from the inherent subjectivity of sentiment analysis tasks, in which everyone assigns a sentiment to a text according to their perspectives [43]. Next, using this annotated database, we verified the performance of each model individually and analyzed the performance of all of the models; Distilled BERT (model 1) performed the best, reaching an accuracy of 80.3%. In addition, the combined model yielded the best results (84% accuracy). It is worthy to note that the addition of RoBERTa (model 2) and BERTweet (model 3) did not improve the accuracy by much, but the addition of fine-tuned RoBERTa (model 5) resulted in a 4% increase in accuracy. Although the overall performance of fine-tuned RoBERTa (model 5) was lower than that of Distilled BERT (model 1), it had higher accuracy (71%) in detecting positive tweets than Distilled BERT (model 1; accuracy: 38.7%; confusion matrices are found in Figure S1 in Multimedia Appendix 2). Therefore, the combined model had superior accuracy in detecting positive tweets (54.8%) compared to Distilled BERT (model 1). Furthermore, based on Table 1, it can be deduced that keeping RoBERTa (model 2) and BERTweet (model 3) in the combination is necessary because the combination of Distilled BERT (model 1) and fine-tuned RoBERTa (model 5) yielded worse results (77.2%). This could be explained by the fact that while fine-tuned RoBERTa (model 5) had better performance in recognizing positive tweets, its performance in recognizing neutral and negative tweets was not very promising; thus, it reduced the overall accuracy. This shows the efficiency of DST in combining the models and exploiting the strength of each model to improve the overall classification of sentiments.

Having developed a reliable pipeline for sentiment analysis, we analyzed the emotions of tweets. During the peripandemic interval, we observed a significantly higher number of tweets with the keyword *insomnia* (*P*<001). A possible explanation is that social interactions shifted from in-person environments to web-based environments, such as Twitter. The number of Twitter’s annual users increased by 33.8%, from 138 million users in 2019 to 186 million users in 2020 [44,45]. We also observed a rise in the total number of insomnia-related tweets after the pandemic began. Considering this, in conjunction with the results of the sentiment analysis, we believe this spike could be related to the rise in negative tweets (Figure 4). According to Table 3, while there was an 8.1% decrease in the number of positive tweets related to insomnia, this number was overshadowed by an 8.3% spike in the number of negative tweets; the number of neutral tweets did not change meaningfully (0.2% decrease). Our findings on the significant increase in the number of negative tweets (*P*<001) during the pandemic is consistent with previously published literature [46]. Politis et al [47] showed an increase in negative sentiment on certain dates by analyzing tweets that were posted before and after the outbreak of the COVID-19 pandemic.

A previous study by Nota and Coles [48] showed that individuals experiencing sleep disruption exhibited diminished top-down inhibitory processes for controlling negative emotions and often engaged in repetitive negative thinking (rumination). We observed the same trend in our study; individuals with insomnia were more prone to rumination when they were awake and free from distractions at night (Figure 5B), suggesting a state of frustration after a poor night of sleep. This corresponds with the observation from Figure 5A, which shows that 62.4% (190,521/305,321) of the negative tweets were generated after midnight.

Our study showed that NLP tools can be used to monitor people’s attitudes toward public stress, such as stress resulting from a pandemic. Policy makers and public health authorities may benefit from using such surveillance tools to better advocate for constituents [49]. Our study is classified as an infodemiology study, which offers an opportunity to analyze public sentiment in real time [50]. NLP tools are strong tools for analyzing and mining Twitter, which is a source of soft intelligence.

### Limitations

In this study, we used Twitter as the source of data collection. As such, we might have excluded a large population that uses other social media platforms (eg, Facebook) or discussion forums (eg, Reddit) to express their perceptions about insomnia. Future studies should investigate publicly available data on other social media platforms in addition to those on Twitter. Further, as this study was based on tweets, it lacks validity measures, as no questionnaires or self-reported measures were used. A future study could use Twitter data and self-reported measures for individuals, health professionals, researchers, and nonprofit organizations in conjunction to assess the needs of pregnant women and the perceived available support and resources during the COVID-19 pandemic.

Of note, in this work, only the keyword *insomnia* was used to scrape the tweets. Although synonyms such as *sleeplessness* could have been used, we were interested only in the clinical term *insomnia*. A study that captures data on the broader area of sleep (ie, beyond insomnia) would be useful for further understanding the full effect of the pandemic. Additionally, several possible confounding factors, such as user location, were not available for all users; such factors may hinder the effect of geolocation on perceptions of insomnia.

### Conclusion

In this study, we proposed a novel NLP pipeline that was based on a combination of transformers using DST to predict the sentiments inherent in text data. We manually annotated 300 tweets and combined various transformer architectures via DST. This combination resulted in higher accuracy for sentiment analysis. By using this pipeline on insomnia-related tweets, our study showed the negative effect of the COVID-19 pandemic on individuals’ experiences of reporting insomnia on Twitter. To investigate the changes in Twitter users’ reported sleep behaviors in the context of the COVID-19 pandemic, we analyzed tweets about insomnia that were posted before and during the pandemic (2019 and 2020). A strength of this study was using NLP and DST to identify tweets about insomnia and analyze their sentiments. In the future, we will assess the effects of changes in other aspects of mental health states (eg, boredom, fear, disgust, surprise, etc) and lifestyle changes (eg, changes in sleep duration, sleep schedules, substance use, physical activity, and sleep medication use) on insomnia symptoms during and after the pandemic based on Twitter and other social media platforms.

## Appendix

Multimedia Appendix 1 A brief theory of transformers.

Multimedia Appendix 2 Supplementary tables and figures.

## Abbreviations

BERT: Bidirectional Encoder Representations From Transformers
DST: Dempster-Shafer theory
NLP: natural language processing
OR: odds ratio
RoBERTa: Robustly Optimized Bidirectional Encoder Representations From Transformers Pretraining Approach
