# Supplementary material for: Sentiment Analysis of Insomnia-Related Tweets via a Combination of Transformers Using Dempster-Shafer Theory: Pre– and Peri–COVID-19 Pandemic Retrospective Study
Source: J Med Internet Res. 2022 Dec 27;24(12):e41517. doi: 10.2196/41517 (PMC9822178; doi:10.2196/41517)
Supplement: Multimedia Appendix 1 [file jmir_v24i12e41517_app1.docx]

Transformers’ Theory:

The building blocks of a Transformer unit are a multihead self-attention mechanism, a position wise feed-forward network, layer normalization modules and residual connectors. Assume the input data as an n-word (tokens) text, which can be defined as:

|  | $X=x_{1}, x_{2}, \ldots x_{n}$ | (1) |
| --- | --- | --- |

This is the input fed to the first layer of the network. The next layer, which is called Positional Embeddings, maps each one-hot token representation into a higher dimension. Consequently, the new tensor is passed through a multiheaded self-attention module. Keys, Values and Queries are an important part of the self-attention module that calculate the attention weights:

|  | $ATTENTION=softmax\left( \frac{QK^{T}}{\sqrt{d_{k}}} \right) V$ | (2) |
| --- | --- | --- |

Where the attention weights are calculated using a softmax function, and $Q$, $K$, and $V$ stand for the queries, keys and values that are basically dot products of the Input with their respective weights:

|  | $Q=XW_{Q}$ | (3) |
| --- | --- | --- |
|  | $K=XW_{K}$ | (4) |
|  | $V=XW_{V}$ | (5) |

The last parameter $d_{k}$ is also known as the scaling factor. There is a direct path from input to the output of the multiheaded self-attention unit, which is called a residual connector; and the output is fed into a normalization layer. Finally, the normalized output of the multiheaded self-attention module is passed to a 2-layered feed-forward network. The inputs/ outputs of this network are similarly connected in a residual fashion with layer normalization.
