# Supplementary material for: Sentiment Analysis of Insomnia-Related Tweets via a Combination of Transformers Using Dempster-Shafer Theory: Pre– and Peri–COVID-19 Pandemic Retrospective Study
Source: J Med Internet Res. 2022 Dec 27;24(12):e41517. doi: 10.2196/41517 (PMC9822178; doi:10.2196/41517)
Supplement: Multimedia Appendix 2 [file jmir_v24i12e41517_app2.docx]

| 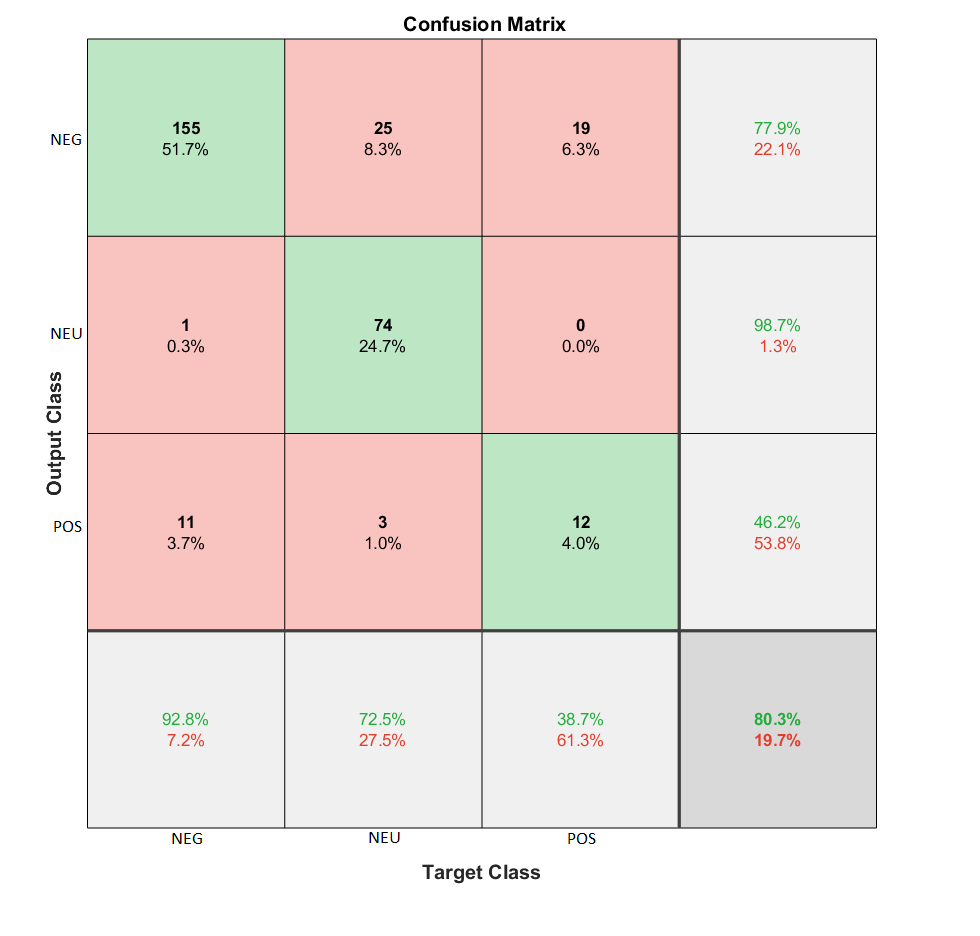  A | 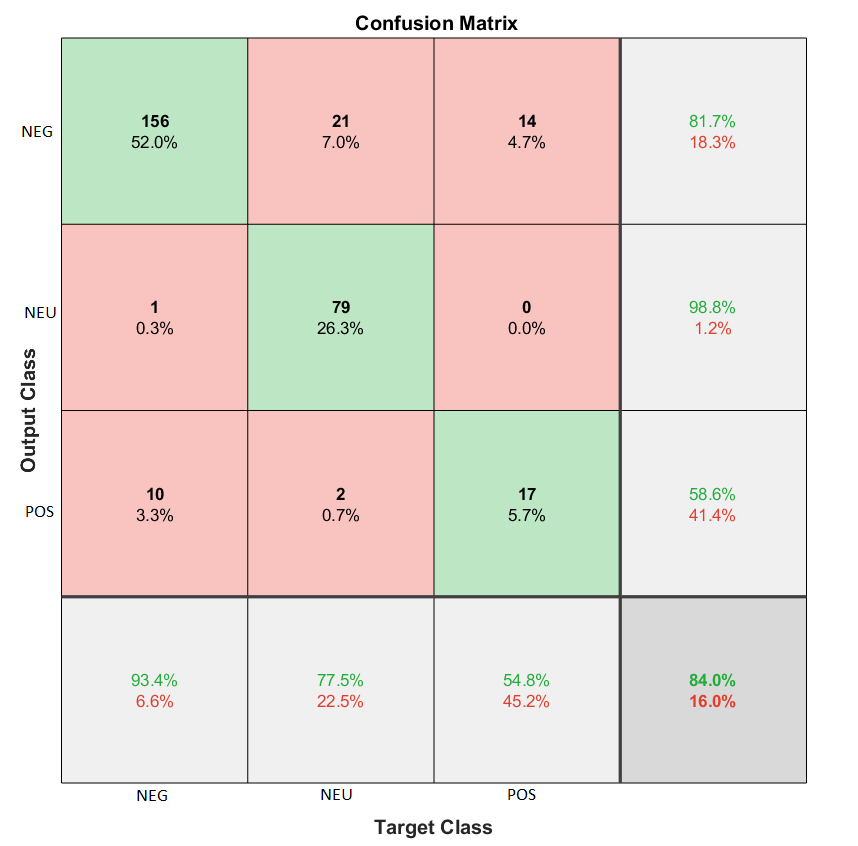  B |
| --- | --- |
| 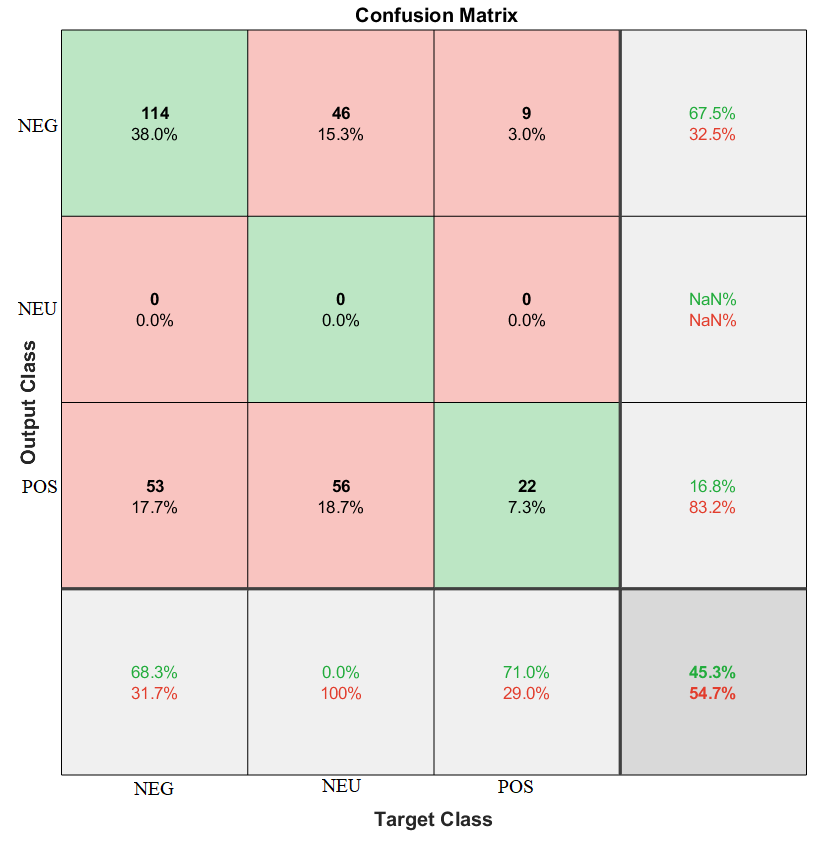  C | |

Figure S1. Confusion matrices for A) Model 1, B) Best DST combination and C) Model 5.


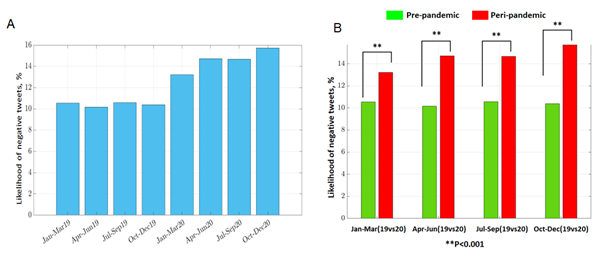


Figure S2. Likelihood of negative tweets in quarterly periods (3 months period) of pre- and peri-pandemic.

| **Table S1. Statistics of likelihood of negative tweets in quarterly (three-month) period.** | | | | |
| --- | --- | --- | --- | --- |
|  | Jan-Mar(19vs20) | Apr-Jun(19vs20) | Jul-Sep(19vs20) | Oct-Dec(19vs20) |
| Odds ratio | 1.22 | 1.12 | 1.11 | 1.12 |
| 95 % CI: | 1.191to 1.26 | 1.09 to 1.16 | 1.09 to 1.15 | 1.09 to 1.15 |
| z statistic | 13.55 | 7.99 | 7.69 | 7.95 |
| Significance level | P < 0.001 | P < 0.001 | P < 0.001 | P < 0.001 |

| **Table S2. Comparison of recent studies on sentiment analysis** | | | | |
| --- | --- | --- | --- | --- |
| Reference/Year | Database | Method | Performance (%) |  |
| [19]/2021 | 6,6149,06 tweets related to 2020 US presidential election | Naive Bayes Classifier | 94.58% accuracy |  |
| [24]/2020 | US Airline (14,640 tweets), Airlines Dataset (16,454 tweets) and Emirates Airline (22,172 tweets) | Transformers | 95% accuracy |  |
| [22]/2019 | 22,821 comments on Wiebo (a Chinese microblogging website) | Transformers | 61.67% accuracy |  |
| [25]/2019 | 582 documents from several financial news sources | Bidirectional Encoder Representations from Transformers (BERT)/ Fine-tuning | 72.5% F1 score |  |
| [21]/2021 | 180,000 COVID19-related tweets provided by Naseem et al [23] | Transformer-based multi-depth distilled BERT | 96.66% accuracy |  |
